# Nrf2 Protein Serum Concentration in Human CKD Shows a Biphasic Behavior

**DOI:** 10.3390/antiox12040932

**Published:** 2023-04-14

**Authors:** Marianne Rasmussen, Kristian Horsman Hansen, Alexandra Scholze

**Affiliations:** 1 Department of Nephrology, Odense University Hospital, 5000 Odense C, Denmark; marianne.rasmussen1@rsyd.dk; 2 OPEN Lab, Odense University Hospital, 5000 Odense C, Denmark; khorsman@health.sdu.dk; 3 Institute of Clinical Research, University of Southern Denmark, 5000 Odense C, Denmark

**Keywords:** chronic kidney disease, Nrf2, Nrf2 activation, oxidative stress, CKD stage, renal failure, redox regulation, inflammation, kidney function, diabetes

## Abstract

Oxidative stress contributes to the progression of chronic kidney disease (CKD) and CKD-related mortality. The nuclear factor erythroid 2-related factor 2 (Nrf2) is essential in the regulation of cellular redox status, and Nrf2-activating therapies are under evaluation in several chronic diseases, including CKD. It is therefore inevitable to understand how Nrf2 behaves in advancing CKD. We analyzed Nrf2 protein concentrations in patients with varying extents of CKD but without renal replacement therapy, and in healthy subjects. Compared to healthy controls, Nrf2 protein was upregulated in mild to moderate kidney function impairment (G1–3). Within the CKD population, we found a significant positive correlation between Nrf2 protein concentration and kidney function (estimated glomerular filtration rate). In severe kidney function impairment (G4,5), Nrf2 protein was reduced compared to mild to moderate kidney function impairment. We conclude that Nrf2 protein concentration in severe kidney function impairment is reduced relative to the mild to moderate kidney function impairment where increased Nrf2 protein concentrations prevail. With respect to the implementation of Nrf2 targeted therapies, it will be necessary to explore in which population of patients with CKD such therapies are able to effectively add to the endogenous Nrf2 activity.

## 1. Introduction

The transcription factor nuclear factor erythroid 2-related factor 2 (Nrf2) is a central component in the regulation of cellular redox status and has gained considerable interest as a therapeutic target in many chronic diseases [1,2]. In chronic kidney disease (CKD), the Nrf2 system plays a crucial role in both CKD progression and the considerable CKD-related morbidity [3,4].

Nrf2 (molecular weight ~95–110 kDa) is ubiquitously expressed and functions as a transcription factor that responds to reactive oxygen species (ROS) production. It belongs to the cap “n” collar subfamily of basic-region leucine zipper transcription factors. Nrf2 binds to DNA in an acidic region, thereby regulating transcriptional activity. The Kelch-like ECH-associated protein 1 (Keap1) is a negative regulator of Nrf2 and mediates its degradation via proteasomes [5]. Cellular ROS production was shown to be increased in CKD [6]. Upon ROS production, Keap1 is oxidized, permitting Nrf2 release and accumulation. Nrf2 translocates to the nucleus and regulates the gene expression of its numerous target genes involved in antioxidant response, inflammation, and lipid and carbohydrate metabolism [7].

In human kidney disease, the Nrf2 system seems to display different functional states relating to the extent of kidney failure and comorbidities [3,4,8]. In kidney disease, endogenous activation and upregulation of the Nrf2 system related to oxidative stress and mild to moderately increased concentrations of the uremic toxin indoxyl sulfate have been described ([9,10,11], reviewed in [4]). Additionally, the uremic toxin methylglyoxal could contribute to an increase in Nrf2 protein concentration through Keap1 cross-linking [12]. On the other hand, there exists broad preclinical evidence from animal models of renal diseases that the Nrf2 pathway was downregulated, both in renal and nonrenal cells [13,14]. So far, in human CKD, Nrf2 has almost exclusively been investigated in patients with advanced disease. Those studies mostly reported Nrf2 repression (systematically reviewed in [15]). 

Preclinical studies showed that pharmacological Nrf2 activation could attenuate kidney injury [16,17,18]. Therefore, there is considerable interest in the development and clinical testing of different classes of Nrf2-activating substances [1,19]. However, it has been highlighted that the context in which Nrf2 is targeted is of utmost importance. This also applies to the leading cause of CKD-related mortality, cardiovascular disease, where both protective and exacerbating effects of Nrf2 have been observed [1]. 

The endogenous status of Nrf2 in human CKD is a key component for the context of potential Nrf2-targeting therapies. Therefore, we investigated Nrf2 protein concentration in stages of progressive kidney failure and compared the results to healthy control subjects. Furthermore, we tested the relation of Nrf2 protein to glomerular filtration rate as a measure of kidney function. 

We found a significant positive association between the glomerular filtration rate and Nrf2 protein concentration in our patient population with CKD. Our results showed a biphasic behavior of Nrf2 protein concentration. While Nrf2 protein was increased in mild to moderate kidney function impairment compared to healthy subjects, we found a significant reduction in Nrf2 protein in severe kidney function impairment.

## 2. Materials and Methods

### 2.1. Study Subjects

We enrolled 75 patients with non-dialysis-dependent CKD from the outpatient clinic of the Department of Nephrology, Odense University Hospital. Patients were approached consecutively as they came to the nephrological outpatient clinic on the days of blood sampling. Inclusion and exclusion criteria were prespecified and uniformly applied to the study participants. The inclusion criteria were being legally competent, age > 18 years, and verified CKD according to the 2012 Clinical Practice Guideline for the Evaluation and Management of Chronic Kidney Disease [20]. CKD stages (G1–G5) were determined based on the eGFR categories accordingly. The exclusion criteria were pregnancy and breastfeeding, critical illness, functioning kidney graft, dialysis therapy, and no written informed consent.

The study enrolled 21 healthy control subjects. These persons were recruited by personal contact among students and staff at the University of Southern Denmark in the same time period as the CKD patients. Inclusion and exclusion criteria were prespecified and uniformly applied to the study participants. The inclusion criteria were being legally competent, age > 18 years, and absence of chronic disease in the medical history. The exclusion criterion was no written informed consent.

The study was approved by the Regional Ethics Committee (S-20110061) and written informed consent was obtained from all study participants before inclusion into the study.

### 2.2. Collection of Clinical Data and Blood Samples

Clinical data were obtained from medical records on actual medical status and medical history. This included the record of age, sex, height, weight, cause of kidney disease, comorbidities, and routine blood biochemical analyses. Venous blood samples were drawn in the morning from study participants. Serum was separated and stored at −80 °C until the batch analysis of samples for Nrf2. The eGFR was calculated using the 2009 CKD-EPI creatinine equation. All biochemical analyses, including the determination of Nrf2 protein were performed by laboratory staff blinded to the clinical characteristics of the study participants, including eGFR categories.

### 2.3. Determination of Nrf2 Protein

Serum concentrations of Nrf2 were measured by enzyme-linked immunosorbent assay (ELISA) using kits from Invitrogen, ThermoFisher Scientific, Göteborg, Sweden (catalogue #EH348RB) that can be utilized to analyze human Nrf2 in serum, plasma, and cell culture supernatants. The kit was run according to the manufacturer’s instructions. The lowest detectable Nrf2 serum concentration was 21 pg/mL. Samples with an Nrf2 concentration below the detection limit (DL) were set to 0.5 DL, hence 10.5 pg/mL in the statistical analyses. This applied to 10 out of 21 healthy control subjects, 8 out of 37 patients with CKD G1–3, and 19 out of 38 patients with CKD G4,5. The coefficient of variation (%CV) of the standards was below 22.6 with an average %CV of 10.8.

### 2.4. Statistics

The data distribution was tested using the D’Agostino and Pearson omnibus normality test. Continuous variables are given as medians and interquartile ranges, and categorical variables are given as counts and percentages. Groups were compared using the Kruskal–Wallis test or the Mann–Whitney test, as appropriate. Differences in categorical variables between groups were analyzed by Fisher’s exact test. A non-parametric bivariate correlation analysis (Spearman) was performed. The relation between a continuous and a binary categorical variable was assessed by comparison of the respective two groups containing the continuous data using the Mann–Whitney test. Data were analyzed using GraphPad prism software (version 5.0; GraphPad Software, San Diego, CA, USA). All statistical tests were two-sided and a two-sided value of *p* < 0.05 was considered statistically significant.

## 3. Results

Clinical and biochemical characteristics of the study subjects are given in Table 1. The CKD population included CKD G1 (2 subjects), CKD G2 (2 subjects), CKD G3 (33 subjects), CKD G4 (32 subjects), and CKD G5 (6 subjects).

We analyzed the relation between Nrf2 protein concentration and clinical parameters that might be related to Nrf2 according to the literature. Table 2 gives the rationale for the parameter selection.

Our analyses showed a significant positive correlation between eGFR and Nrf2 protein concentration in the CKD population in our study (n = 75; Spearman r = 0.30, *p* = 0.009). We did not detect a significant association of Nrf2 protein with the presence of diabetes, proteinuria, and C-reactive protein. A summary of the relations of Nrf2 protein to clinical parameters is given in Table 3.

We then compared Nrf2 serum protein concentration between healthy control subjects, patients with mild to moderate kidney function impairment (G1–3), and severe kidney function impairment (G4,5). Table 4 shows the clinical characteristics of the two CKD populations, which differed significantly with respect to their glomerular filtration rate.

**Figure 1:**
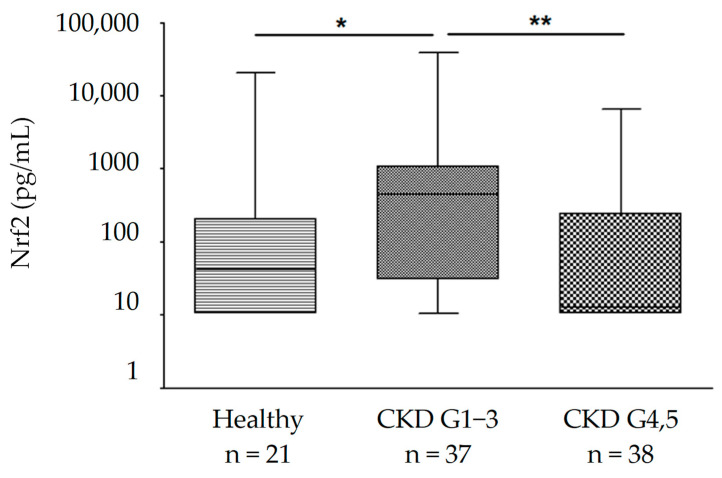
Nuclear factor erythroid 2-related factor 2 (Nrf2) protein concentrations in healthy control subjects, mild to moderate, and severe kidney function impairment. Nrf2 protein concentration in serum was determined by ELISA. The values are shown as box and whiskers (minimum to maximum). The Nrf2 protein concentration was significantly higher in patients with mild to moderate kidney function impairment compared to healthy subjects (Dunn’s post test * *p* < 0.05) and patients with severe kidney function impairment (Dunn’s post test ** *p* < 0.01).

Nrf2 protein concentrations between the three groups differed significantly (Kruskal–Wallis *p* = 0.003). As shown in Figure 1, Nrf2 was significantly higher in patients with mild to moderate kidney function impairment compared to both the patients with severe kidney function impairment and the healthy control subjects. The protein concentrations from healthy subjects and patients with CKD 4,5 did not significantly differ.

The median Nrf2 protein concentration was 42.4 pg/mL (interquartile range 10.5–204.8 pg/mL) in healthy control subjects, 454.4 pg/mL (interquartile range 30.9–1082 pg/mL) in mild to moderate kidney function impairment, and 12.8 pg/mL (interquartile range 10.5–247.4 pg/mL) in severe kidney function impairment.

From our results, we conclude that in the population of patients with CKD a significant positive association between glomerular filtration rate and Nrf2 protein exists. Compared to healthy controls, an upregulation of Nrf2 protein was found in mild to moderate kidney function impairment, while the Nrf2 protein concentration in severe kidney function impairment (CKD 4,5) was significantly lower compared to less advanced CKD stages.

## 4. Discussion

We report a biphasic behavior of Nrf2 protein concentration in CKD. In our study, mild to moderate kidney function impairment was characterized by higher Nrf2 protein concentration than in healthy subjects. As lower glomerular filtration rates were associated with lower Nrf2 protein concentration within the CKD population, Nrf2 protein concentration in severe kidney function impairment was significantly reduced compared to mild to moderate kidney function impairment. Nrf2 protein concentration was comparable between CKD G4,5 and healthy subjects. The latter result is in line with findings on Nrf2 gene expression in peripheral blood mononuclear cells reported from patients with CKD G3–5 [26]. On the other hand, a small but significant decrease in Nrf2 protein in plasma from CKD G4,5 compared to healthy controls has been reported [27]. Our finding that Nrf2 protein concentration was significantly reduced in more advanced CKD compared with less advanced CKD is supported by results from plasma analyses [28] and kidney biopsies [29]. Such reduction in the Nrf2 protein could occur due to the increasing concentration of the uremic toxin indoxyl sulfate, which has been reported to reduce the Nrf2 gene and protein expression at high concentrations [30,31].

The higher Nrf2 protein concentration, seen in our study in mild to moderate kidney function impairment, might be expected, as the Nrf2 accumulation with adjacent translocation to the nucleus and induction of antioxidant target genes is the adequate response to an oxidative challenge to sustain or regain redox homeostasis [5]. Such upregulation of the Nrf2 system related to oxidative stress was described in kidney tissue samples from patients with Lupus nephritis and patients with diabetic kidney disease [9,10]. In addition, a significant increase in gene expression of the Nrf2 target gene NAD(P)H quinone dehydrogenase 1 (NQO1) was reported in monocytes from patients with CKD without renal replacement therapy [32].

An interesting point is the high number of samples with very low serum Nrf2 protein concentrations, below the limit of detection, in both the healthy control subjects and in patients with very advanced CKD. While in healthy subjects this might reflect lower requirement for stimulation of the Nrf2 system, in patients with very advanced CKD this could hint to a reduced capacity for Nrf2 system activation.

We also investigated the relation between Nrf2 protein and clinical characteristics in our CKD population. The relation between the presence of diabetes and Nrf2 protein concentrations was analyzed since a significantly higher plasma concentration of the Nrf2 target NQO1 had been reported in plasma from diabetic patients [25], pointing to higher Nrf2 activity in diabetes. Furthermore, the activation of Nrf2 had been shown to prevent biochemical dysfunctions induced by hyperglycemia [33]. Therefore, a compensatory increase in Nrf2 in diabetes might be plausible. Nevertheless, in our population of patients with CKD a relation between Nrf2 protein concentration and the presence of Diabetes mellitus was not observed, maybe due to the relatively low prevalence of diabetes in our patient population.

Another important parameter with respect to Nrf2 is proteinuria. A positive correlation between the Nrf2 target heme oxygenase 1 (HO-1) and proteinuria has been reported [24], and in a mouse model, Nrf2 activation increased proteinuria [34]. In our patient population, a relation between the extent of proteinuria and Nrf2 protein was not observed, but this issue should be addressed in a population with similar eGFR and varying severity of proteinuria.

The significant interplay between inflammation and oxidative stress in CKD has been stressed [35,36,37,38]. Our analyses did not show a significant correlation between the inflammation marker C-reactive protein and Nrf2 protein concentration. The relationship between Nrf2 and inflammation is complex, with reported Nrf2 induction through inflammatory stimuli and Nrf2-mediated suppression of inflammation [4,39]. It might, therefore, not be possible to detect the relation between Nrf2 and inflammatory parameters in an organism by simple correlation analysis, as long as Nrf2-related mechanisms are an adequate response to stress conditions.

An important question that arises from our study is how the consequences of both the upregulation of Nrf2 in mild to moderate kidney function impairment and the downregulation of Nrf2 in severe kidney function impairment can be assessed. We suggest that it is necessary to perform prospective cohort studies that investigate CKD progression and CKD-related morbidity in relation to Nrf2 concentration. To assess Nrf2 efficiency in these prospective studies, measurements of Nrf2 concentration and markers of oxidative damage should be combined. Such analyses could guide the design of clinical studies with Nrf2-activating substances in CKD subgroups defined by CKD stage and comorbidities.

An assessment of plasma Nrf2 protein as a surrogate for Nrf2 protein concentration in different cell types and tissues needs to be established in human CKD. On the one hand, factors such as systemic inflammation, uremic toxins or comorbidities exert their effects on all cell types. We recently found, in a systematic review, that the state of Nrf2 system components depended mainly on CKD stage, comorbidities, and investigated Nrf2 target rather than investigated cell type [15]. On the other hand, each cell type or tissue can possess its own additional pathomechanisms that regulate the Nrf2 system in either direction; optimal monitoring strategies are yet to be developed [40].

Study limitations: The low number of study participants is a limitation of our study. Furthermore, a biochemical characterization of healthy study participants would be advantageous. In addition, healthy control subjects were significantly younger and had a lower BMI compared to patients with CKD. We suggest that future studies be performed to reinforce our results, especially with respect to an analysis of Nrf2 protein concentration separately for each stage of CKD.

## 5. Conclusions

In conclusion, we found that Nrf2 protein concentration in severe kidney function impairment was reduced relative to the mild to moderate kidney function impairment. In the earlier stages of CKD with mild to moderately reduced kidney function (CKD G1–3), Nrf2 protein concentrations were increased compared to healthy control subjects. With respect to the implementation of Nrf2 targeted therapies, it will be important to analyze in which CKD stages or kidney diseases Nrf2-activating or Nrf2-modifying therapies can effectively support the endogenous Nrf2 activity and/or Nrf2 targets.

## Figures and Tables

**Table 1:** Clinical and biochemical population characteristics of patients with chronic kidney disease and healthy control subjects, given as median (25–75% percentile) or number (percentage).

	Healthy (n = 21)	CKD (n = 75)
Age, years	35 (30–45) *	67 (57–75)
Men, n (%)	12 (57) #	46 (61)
BMI, kg/m^2^	23.8 (22.3–25.7) ꙳	27.8 (24.0–35.7)
Kidney disease, underlying cause, n (%) Glomerulonephritis Diabetic nephropathy Hypertensive nephropathy Hereditary Other/unknown	None	17 (23) 2 (3) 5 (7) 4 (5) 47 (63)
Diabetes, n (%)	None	16 (21)
Hypertension, n (%)	None	65 (87)
eGFR, mL/min/1.73 m^2^	n.d.	30 (19.0–42.0)
Urea (mmol/L)	n.d.	13.1 (9.7–18.3)
Proteinuria (g/d)	n.d.	0.63 (0.2–2.6)
CRP (mg/L)	n.d.	4.0 (2.0–9.0)

Abbreviations: CKD-chronic kidney disease; BMI-body mass index; eGFR-estimated glomerular filtration rate; n.d.-not done; CRP-C-reactive protein; comparisons healthy vs. CKD: * *p* < 0.0001, ꙳ *p* < 0.01, # n.s.

**Table 2:** Basis for the selection of clinical parameters for the relation analyses with Nrf2 protein concentration in our study.

	Reported Relation to Nrf2	Reference
eGFR	Impaired Nrf2 activity contributed to the progression of kidney damage	[21]
Proteinuria	Increased proteinuria in clinical studies with pharmacological Nrf2 activation	[22]
CRP	Nrf2 activators have demonstrated anti-inflammatory activity	[23]
Diabetes	Increased expression of Nrf2 target genes HO 1 and NQO1 in patients with diabetes	[24,25]

Abbreviations: Nrf2-Nuclear factor erythroid 2-related factor 2; eGFR-estimated glomerular filtration rate; CRP-C-reactive protein.

**Table 3:** Relations between Nrf2 protein concentration and selected clinical parameters in patients with CKD (n = 75).

	Spearman r	*p*
eGFR	0.30	<0.01
Proteinuria	0.03	0.86
CRP	−0.12	0.29
Diabetes Yes/No (binary categorical variable)		Mann–Whitney *p* 0.32

Abbreviations: eGFR-estimated glomerular filtration rate; CRP-C-reactive protein.

**Table 4:** Comparison of clinical parameters with potential relation to Nrf2 between mild to moderate kidney function impairment (G1–3) and severe kidney function impairment (G4,5) CKD. Values are given as median (25–75% percentile) or number (percentage).

	CKD G1–3 (n = 37)	CKD G4,5 (n = 38)	Mann–Whitney *p*
age	66.0 (55.5–71.5)	68.0 (58.8–78.3)	0.07
BMI	30.1 (23.5–38.6)	27.3 (24.7–33.1)	0.72
eGFR, mL/min/1.73 m^2^	42.0 (35.0–53.5)	19.5 (16.0–24.3)	<0.0001
Proteinuria (g/d)	0.5 (0.2–2.6)	1.4 (0.3–3.2)	0.38
CRP (mg/L)	3.7 (1.9–7.3)	5.1 (2.1–12.5)	0.32
			Fisher’s exact *p*
Diabetes, n (%)	5 (14)	11 (29)	0.16

Abbreviations: CKD-chronic kidney disease; eGFR-estimated glomerular filtration rate; CRP-C-reactive protein.

## Data Availability

Due to ethical and data privacy restrictions, the data cannot be deposited in the public domain. Data can be made available upon request, pending ethical approval and a data processing agreement with the Region of Southern Denmark.
